# Function approximation approach to the inference of reduced NGnet models of genetic networks

**DOI:** 10.1186/1471-2105-9-23

**Published:** 2008-01-14

**Authors:** Shuhei Kimura, Katsuki Sonoda, Soichiro Yamane, Hideki Maeda, Koki Matsumura, Mariko Hatakeyama

**Affiliations:** 1 Faculty of Engineering, Tottori University, 4-101 Koyama-Minami, Tottori, Japan; 2 JFE R&D Corporation, 1-1 Minami-Watarida, Kawasaki, Japan; 3 JFE Engineering Corporation, 2-1 Suehiro, Tsurumi, Yokohama, Japan; 4 RIKEN Genomic Sciences Center, 1-7-22 Suehiro, Tsurumi, Yokohama, Japan

## Abstract

**Background:**

The inference of a genetic network is a problem in which mutual interactions among genes are deduced using time-series of gene expression patterns. While a number of models have been proposed to describe genetic regulatory networks, this study focuses on a set of differential equations since it has the ability to model dynamic behavior of gene expression. When we use a set of differential equations to describe genetic networks, the inference problem can be defined as a function approximation problem. On the basis of this problem definition, we propose in this study a new method to infer reduced NGnet models of genetic networks.

**Results:**

Through numerical experiments on artificial genetic network inference problems, we demonstrated that our method has the ability to infer genetic networks correctly and it was faster than the other inference methods. We then applied the proposed method to actual expression data of the bacterial SOS DNA repair system, and succeeded in finding several reasonable regulations. When our method inferred the genetic network from the actual data, it required about 4.7 min on a single-CPU personal computer.

**Conclusion:**

The proposed method has an ability to obtain reasonable networks with a short computational time. As a high performance computer is not always available at every laboratory, the short computational time of our method is a preferable feature. There does not seem to be a perfect model for the inference of genetic networks yet. Therefore, in order to extract reliable information from the observed gene expression data, we should infer genetic networks using multiple inference methods based on different models. Our approach could be used as one of the promising inference methods.

## Background

With recent advances in technologies such as DNA microarrays, it has become possible to measure gene expression patterns on a genomic scale. One expected use of these data is to predict functions of genes through the inference of regulatory interactions of genes, i.e., a genetic network. There are increasing needs to reveal unknown functions of genes. Therefore, many researchers have become interested in the inference of genetic networks, and the development of this methodology has become a major topic in the bioinformatics field.

Numerous models to describe genetic networks have been proposed [1-10]. This study however focuses especially on a set of differential equations since it has an ability to capture dynamic behavior of gene expression. In the genetic network inference problem based on the set of differential equations, a genetic network is described as

(1) $$\begin{array}{cc} \frac{dX_{n}}{dt}=G_{n}(X_{1},\cdots ,X_{N}), & (n=1,\cdots ,N), \end{array}$$

where *X*_*n *_is the expression level of the *n*-th gene, *N *is the number of genes in the network, and *G*_*n *_is a function of an arbitrary form. The purpose of the genetic network inference problem based on the set of differential equations is to approximate the function *G*_*n *_from the observed gene expression data. The function *G*_*n *_is generally approximated using a model of the fixed form; most typically a linear model [7,11,12] or an S-system model [13]. The computational time for inferring linear models of genetic networks is very short. However, the linear model is not suitable for analyzing time-series of gene expression data because it requires that the system operates near a steady state [7]. The S-system model, on the other hand, possesses some properties inherent in biochemical systems. Moreover, several methods are available for analyzing the model. Because of these advantages, a number of inference methods based on the S-system model have been proposed [14-21]. However, some of them are time-consuming because they require solving a set of differential equations many times.

To overcome the shortcomings of the fixed model approaches, we defined the genetic network inference problem as a function approximation problem [10]. For solving the defined problem, any type of function approximator is available. When we use a powerful function approximator to solve this problem, we can obtain a good approximation of the function *G*_*n*_. Therefore, on the basis of this problem definition, we have proposed inference methods that use powerful function approximators, i.e., a neural network model [10], a Normalized Gaussian network (NGnet) model [9], and a reduced NGnet model [22], respectively. As inference methods based on this problem definition do not always require solving any differential equations, our methods needed a low computational effort.

Inference methods based on the function approximation problem, on the other hand, require estimating differential coefficients of the gene expression level before inferring the genetic network. We must estimate them correctly in order to obtain reasonable network models, and a number of techniques are available for this purpose [8,15,17,23]. However, as the measurement data are generally polluted by noise, it is difficult for us to estimate the differential coefficients in advance. This study therefore proposes a new method that performs the inference of the genetic network and the estimation of the differential coefficients simultaneously. Our method uses the reduced NGnet model to describe genetic networks, since it requires a quite low computational effort. Through numerical experiments, we verify the effectiveness of the proposed inference method.

## Results and Discussion

### Inference of an S-system network

In this experiment, we confirmed that, when a sufficient amount of noise-free data are given, the proposed method has an ability to infer the genetic network correctly.

#### Experimental setup

**Figure 1:**
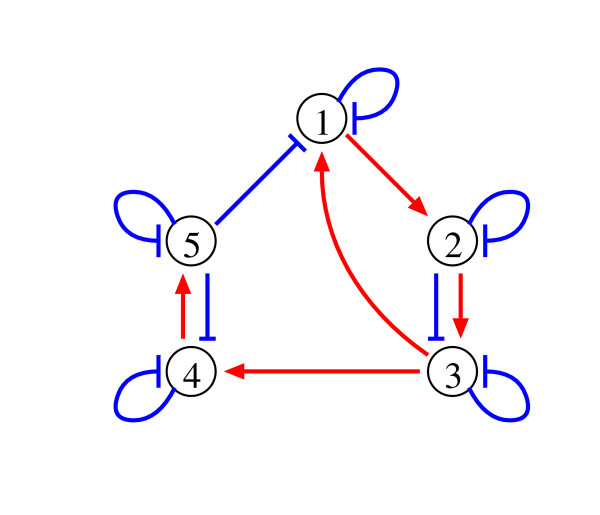
The network structures of the target model. Red lines: positive regulations. Blue lines: negative regulations.

As a target network that we attempt to infer, we used a small-scale S-system model consisting of 5 genes (*N *= 5). The S-system model is often used to describe biochemical networks [8,14,15,18-20,24-26]. The model is structured as a set of non-linear differential equations of the form

(2) $$\begin{array}{r} \frac{dX_{n}}{dt}=\alpha_{n}\prod_{m=1}^{N}X_{m}^{g_{n,m}}-\beta_{n}\prod_{m=1}^{N}X_{m}^{h_{n,m}},\\ (n=1,\cdots ,N), \end{array}$$

where *X*_*n *_is the expression level of the *n*-th gene, *α*_*n *_and *β*_*n *_are multiplicative parameters called rate constants, and *g*_*n*,*m *_and *h*_*n*,*m *_are exponential parameters called kinetic orders. The S-system parameters of the target genetic network are shown in Table 1[16,27]. This study assumes that the *m*-th gene positively regulates the *n*-th gene when *X*_*m *_promotes the synthesis or suppresses the degradation of *X*_*n*_. Similarly, when *X*_*m *_suppresses the synthesis or promotes the degradation of *X*_*n*_, we assume that the *n*-th gene is negatively regulated by the *m*-th gene. When the *m*-th gene positively regulates the *n*-th gene, *g*_*n*,*m *_is positive and/or *h*_*n*,*m *_is negative in the S-system model. When the *m*-th gene negatively regulates the *n*-th gene, *g*_*n*,*m *_is negative and/or *h*_*n*,*m *_is positive. When the *m*-th gene has no influence on the *n*-th gene, the parameters *g*_*n*,*m *_and *h*_*n*,*m *_are zero. Thus, we can illustrate the structure of the target network, as shown in Figure 1.

**Table 1:** The S-system parameters of the target model.

*i*	*α*_*i*_	*g*_*i*,1_	*g*_*i*,2_	*g*_*i*,3_	*g*_*i*,4_	*g*_*i*,5_	*β*_*i*_	*h*_*i*,1_	*h*_*i*,2_	*h*_*i*,3_	*h*_*i*,4_	*h*_*i*,5_
1	5.0	0.0	0.0	1.0	0.0	-1.0	10.0	2.0	0.0	0.0	0.0	0.0
2	10.0	2.0	0.0	0.0	0.0	0.0	10.0	0.0	2.0	0.0	0.0	0.0
3	10.0	0.0	-1.0	0.0	0.0	0.0	10.0	0.0	-1.0	2.0	0.0	0.0
4	8.0	0.0	0.0	2.0	0.0	-1.0	10.0	0.0	0.0	0.0	2.0	0.0
5	10.0	0.0	0.0	0.0	2.0	0.0	10.0	0.0	0.0	0.0	0.0	2.0

As the observed gene expression patterns, fifteen sets of noise-free time-series data, each covering all five genes, were given. The sets began from randomly generated initial values in [0.0, 2.0] and were obtained by solving the differential equations (2) on the target model. In a practical application, these sets of time-series data could be obtained by actual biological experiments. Eleven sampling points for the time-series data were assigned to each gene in each set. Thus, the observed time-series data for each gene consisted of 15 × 11 = 165 sampling points. We inferred the reduced NGnet models solely from these time-series data, and then, extracted interactions between genes from the obtained models. As one reduced NGnet model corresponds to one gene, we must infer 5 models to solve the artificial problem defined here. We carried out 10 trials by changing the seed for the pseudo random number to obtain each model. According to the preliminary experiments (see Additional file 1), we used the following parameters; the weight parameter used in the prior probability distribution *γ *was 20, the maximum indegree *I *was 5, and the number of the units of the reduced NGnet model *M *was 3. We can change the function approximation capability of the reduced NGnet model using the number of units *M*. However, we cannot use the model with an unduly large *M*, since the large *M *makes the estimation problem of the model parameters difficult. An unduly small *M*, on the other hand, should make the model insufficient to represent complicated interactions between genes.

#### Result

**Figure 2:**
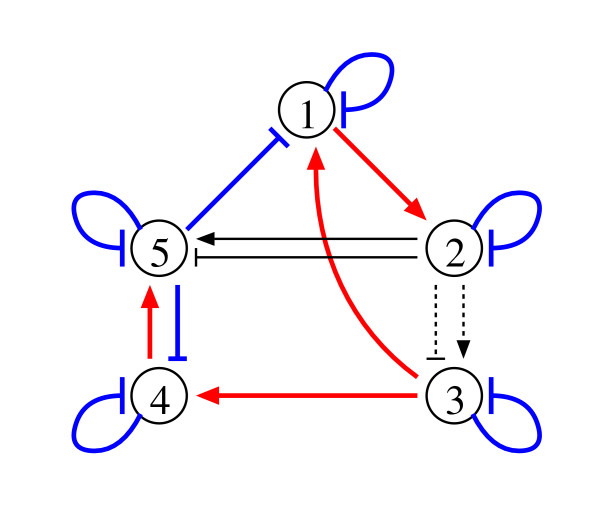
A sample of the network structure inferred by the proposed method. Colored bold lines: true-positive regulations. Thin lines: false-positive regulations. Dotted lines: false-negative regulations.

We extracted interactions between genes from the reduced NGnet models obtained using the method based on the sensitivity analysis [10] (see also the *Method *section). Figure 2 shows a typical genetic network inferred from the obtained models. As the figure illustrates, most of the regulations were correctly inferred by the proposed method. Our method inferred an average of 1.3 ± 1.7 unnecessary regulations that were absent in the target network, i.e., false-positive regulations, and failed to infer an average of 1.9 ± 0.5 regulations that were present in the target network, i.e., false-negative regulations. The sensitivity and the specificity of the proposed method were therefore 0.854 ± 0.041 and 0.967 ± 0.045. respectively. These measures are defined as

(3) $$(\text{sensitivity})=\frac{TP}{TP+FN},$$

(4) $$(\text{specificity})=\frac{TN}{FP+TN},$$

where *TP*, *FN*, *TN *and *FP *are the numbers of true-positive, false-negative, true-negative and false-positive regulations, respectively. The sensitivity increases from 0 to 1 with decreasing the number of false-negative regulations, and the specificity increases from 0 to 1 with decreasing the number of false-positive regulations. The sum of the squared error between the time-course produced by the obtained model and the given time-series data, i.e., the value of the objective function (16) defined in the *Methods *section, was 1.57 × 10^-1 ^± 1.23 × 10^-1 ^on average.

Although our method has an ability to infer the positive and negative regulations of the *n*-th gene from the *m*-th gene simultaneously, it failed in inferring these regulations of the 3rd gene from the 2nd gene in most of the trials. Given that *X*_2 _works to suppress both the synthesis and the degradation of *X*_3 _with the same kinetic order in the target model, we know that the 2nd gene has only a weak impact on the 3rd gene. Therefore, it should be difficult for our method to infer these regulations. In order to infer these difficult regulations correctly, we should give more sets of the gene expression data [15,28].

While the proposed method was unable to infer the target network with perfect precision as mentioned above, its computational time was sufficiently short. The computational time to obtain one reduced NGnet model averaged about 60.8 sec on a single-CPU personal computer (Pentium IV 2.8 GHz). The proposed method therefore required about 60.8 sec × 5 ≃ 5.1 min to solve this genetic network inference problem. In order to infer the same network, on the other hand, PEACE1 [16], the coevolutionary method [18], the method with a collocation approximation [21] and the decoupling method [15] reportedly took about 10 h on a PC cluster (Pentium III 933 MHz × 1040 CPUs), 89.0 min on a PC cluster (Pentium III 933 MHz × 8 CPUs), 2.84 h on a single-CPU personal computer (Pentium IV 2.4 GHz) and 6.38 min on a single-CPU personal computer, respectively. The comparison results present that the proposed method was faster than the other inference methods. As a high performance computer, such as a PC cluster, is not always available at every laboratory, the shorter computational time of the proposed method should be a preferable feature. 

As mentioned before, the proposed method performs the inference of the reduced NGnet model and the estimation of the differential coefficients of the gene expression level simultaneously. When we can estimate the differential coefficients of the gene expression level correctly before inferring the genetic network, however, the proposed method can omit the simultaneous estimation of the differential coefficients of the gene expression level. When the simultaneous estimation of the differential coefficients is omitted, our method is almost equivalent to the inference method proposed in the paper [22]. As the data used in this section contained no noise, the inference ability of our method was not degraded even when the simultaneous estimation of the differential coefficients was omitted. The sensitivity and the specificity of the proposed method without the simultaneous estimation of the differential coefficients of the gene expression level were 0.854 ± 0.041 and 0.961 ± 0.044, respectively. Moreover, the omission of the simultaneous estimation of the differential coefficients made the computational time much shorter. The method without the simultaneous estimation of the differential coefficients required about 2.0 × 5 ≃ 10.0 sec to solve this problem. However, the simultaneous estimation of the differential coefficients improved the sum of the squared error between the time-courses produced by the obtained model and the given time-series data. Thus, the averaged objective values (16) of the method with and without the simultaneous estimation of the differential coefficients were 1.57 × 10^-1 ^± 1.23 × 10^-1 ^and 3.37 × 10^-1 ^± 1.93 × 10^-1^, respectively. These results indicate that, although the simultaneous estimation of the differential coefficients of the gene expression level, proposed in this study, makes the computational cost higher, it produces the models that are suitable for the computational simulation.

### Inference of a random genetic network

Next, we checked the performance of our method in a real-world setting by conducting the experiment with noisy time-series data.

#### Experimental setup

In this experiment, we used the following set of differential equations to describe target networks [7,10].

(5) $$\begin{array}{r} \frac{dX_{n}}{dt}=-\lambda_{n}X_{n}+\frac{\alpha_{n}+\sum_{m\in A_{n}}X_{m}^{\gamma_{n,m}}}{1+\sum_{m\in A_{n}}X_{m}^{\gamma_{n,m}}+\sum_{l\in {ℛ}_{n}}X_{l}^{\beta_{n,l}}},\\ (n=1,\cdots ,N), \end{array}$$

where *λ*_*n *_and *α*_*n *_are the degradation rate and the synthesis rate, respectively, of the *n*-th mRNA, and *γ*_*n*,*m *_and *β*_*n*,*l *_are the activation cooperativity and the repression cooperativity, respectively, of the *m*-th gene on the *n*-th gene. The sets $A_{n}$ and ${ℛ}_{n}$ specify the genes that activate and repress the *n*-th gene, respectively. As the target networks, we randomly constructed the systems of 10, 20 and 30 genes (*N *= 10, 20 and 30). Since the inference ability of the proposed method may depend on the structure of the target network, we changed the network structure on every trial. We generated the target networks of different structures by changing the model parameters described above. In order to-construct the sets $A_{n}$ and ${ℛ}_{n}$, we randomly chose an integer *k *from a power-law distribution with a cutoff 5. Then, *k *genes were randomly selected from all of the genes contained in the network. Finally, the indices corresponding to the selected genes were added to the set $A_{n}$ or the set ${ℛ}_{n}$ with the probability 0.5. The model parameters (*λ*_*n*_, *α*_*n*_, *γ*_*n*,*m *_and *β*_*n*,*l*_) are randomly selected from a uniform distribution.

Since the performances of inference methods generally depend on the amount of given time-series data, we performed the experiments with different numbers of sets of time-series data. We obtained the sets of time-series data by solving the set of differential equations (5). Each set consisted of the expression levels at the eleven time points. The measurement noise was simulated by adding 10% Gaussian noise to the computed time-series data. All of the other experimental conditions were the same as those used in the previous experiment.

#### Results

**Figure 3:**
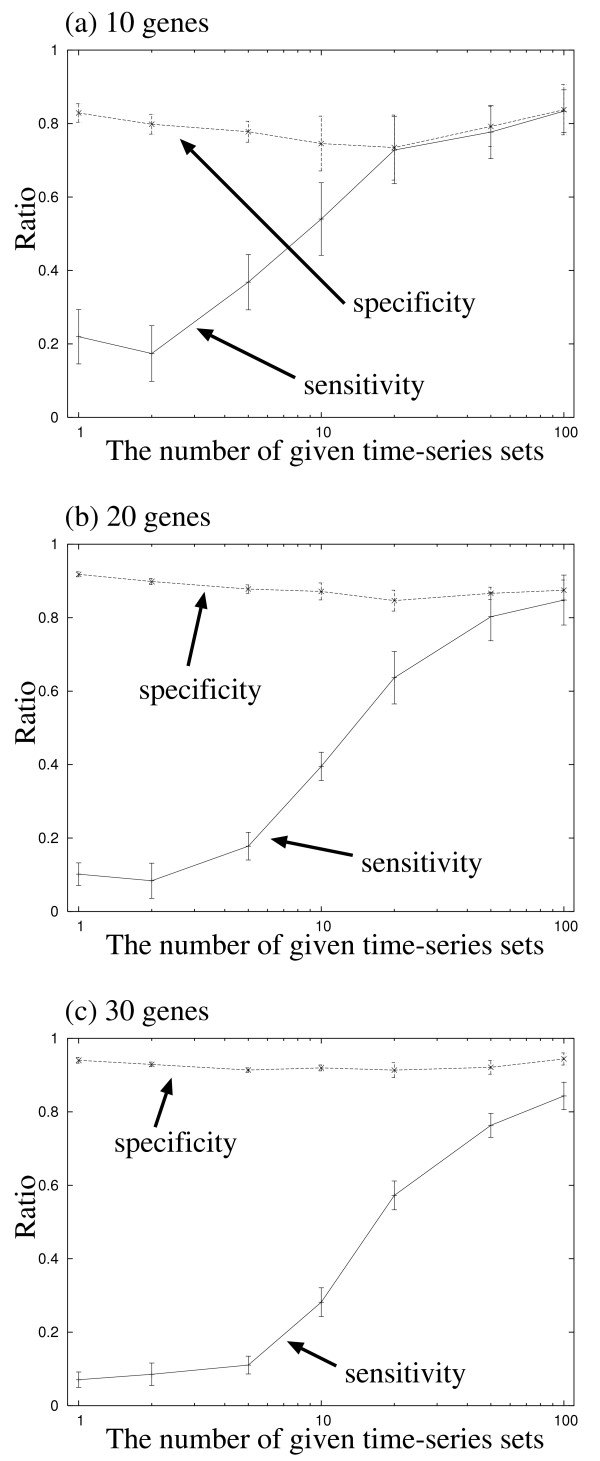
The performances of the proposed method on the experiments of random genetic networks consisting of (a) 10 genes, (b) 20 genes, and (c) 30 genes, respectively. Solid line: the sensitivity. Dotted line: the specificity.

Figure 3(a), (b) and 3(c) show the sensitivity and the specificity of the proposed method on the experiments of the target networks consisting of 10, 20 and 30 genes, respectively. The figures show that the sensitivity of our method is improved as the amount of given time-series data increases. Therefore, to infer genetic networks correctly, we should give the sufficient amount of observed time-series data. As shown in the figures, when we try to achieve a satisfactory level of the sensitivity in a larger-scale problem, we should give a larger amount of observed data. The number of required time-series sets, however, seems not to be proportional to the number of genes contained in the network. This disproportion may be caused by the sparseness of the target network. Therefore, the proposed method should not always require an immense number of time-series sets even when we try to infer large-scale genetic networks.

The specificity, on the other hand, seems to be independent from the amount of given time-series data. When we try to improve it, we must reduce the number of false-positive regulations contained in the inferred model. It is however difficult to reduce these regulations because the maximum indegree *I *used in the prior probability distribution (15) forgives our method for finding several false-positive regulations. Therefore, we should not expect the improvement of the specificity even when we give a larger amount of the observed data.

### Inference of an actual genetic network

Finally, we tested the effectiveness of the proposed method on a genetic network inference problem using actual biological data.

#### Experimental setup

**Figure 4:**
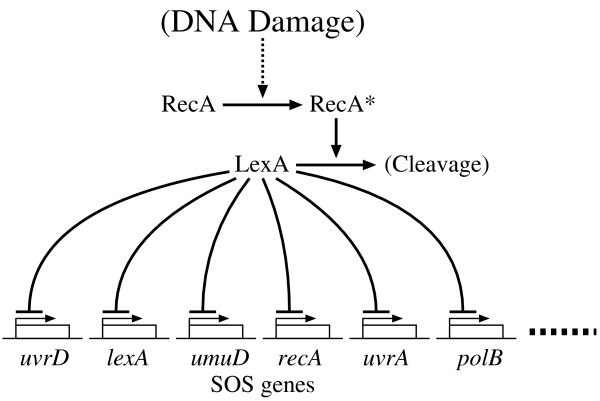
The SOS DNA repair system in *E. coli*.

In this experiment, we used the proposed method to analyze the SOS DNA repair system in *E. coli *(Figure 4) [29]. About 30 genes are known to be involved in this system. In a normal state, a master repressor, LexA, is bound to the interaction sites in the promoter regions of these genes. When DNA damages occur, one of the SOS proteins, RecA, becomes activated and, then, mediates LexA autocleavage. The drop in LexA level causes the de-repression of the SOS genes. Once damage has been repaired, the level of activated RecA drops, LexA accumulates and represses the SOS genes, and the cells return to their original state. We applied the proposed method to the expression data of this system, that were collected by Ronen and his colleagues [30,31]. Then, Cho and his colleagues chose 6 genes from these data and successfully inferred the regulatory network of the selected genes [14]. Therefore, this study also selected the same genes, i.e., *uvrD*, *lexA*, *umuD*, *recA*, *uvrA *and *polB*. Although the data contain 4 sets of time-series data, we used only 2 sets (the third and fourth sets) that were measured under the same experimental condition (see Additional file 1). Each set of the time-series data consists of 50 measurements including the initial concentrations which are all zeros. We, however, removed the initial concentrations from both of the sets, since our model cannot produce different time-courses from the same initial conditions. As it is difficult to calculate an output of the reduced NGnet model against large input values, data corresponding to each gene were normalized against their maximum value. Since the target network contained a small number of the genes, we set the maximum indegree *I *to 3. All of the other experimental conditions were the same as those used in the previous experiments.

#### Results

**Figure 5:**
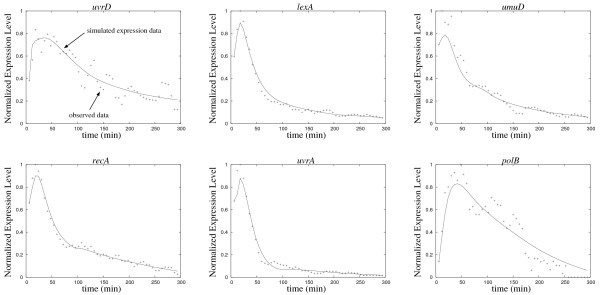
Samples of the time-courses computed from the obtained models on the experiment of the SOS DNA repair system (solid line). The plus symbols are the observed gene expression data.

**Figure 6:**
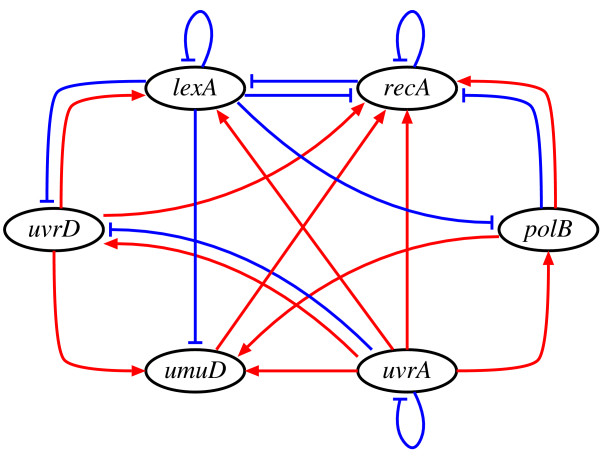
The core network structure inferred by the proposed method.

We succeeded in finding models that simulate the gene expression of the target system well. A sample of the gene expression calculated from the obtained models is shown in Figure 5. Although the network structures inferred by the proposed method were slightly different from each other in 10 trials, most of the regulations were commonly inferred. Figure 6 shows the core network structure where the regulations were inferred more than 7 times within 10 trials. While the inferred networks contained an average of 26.7 ± 3.3 regulations, the core network contained 21 regulations.

The core network contained some reasonable regulations. As Figure 6 shows, although the proposed method failed to infer the regulation from *lexA *to *uvrA*. the negative regulations from *lexA *to the other genes were successfully inferred. As described before, RecA is known to regulate LexA. Though this is a regulatory interaction between proteins, it should be represented by the regulation of *lexA *from *recA *in our network. A number of genes take part in repairing DNA damages, and the accomplishment of DNA repair makes RecA stop the system. The inferred regulations of *recA *from all of the genes might explain this mechanism.

The number of the regulations inferred by the proposed method was larger than that of the S-tree based method proposed by Cho and his colleagues [14]. Although some of our inferred regulations that have not been experimentally observed might be new findings, the rest should be false-positive. In order to infer a more reliable network, we must give more sets of the expression data obtained from additional biological experiments or a priori knowledge about the target system. The computational time of the proposed method was, on the other hand, much shorter. While the S-tree based method running on the computer system (Athlon MP2800+) reportedly took about 35 h to infer the network of this system, the proposed method required about 47.1 sec × 6 ≃ 4.7 min on a single-CPU personal computer (Pentium IV 2.8 GHz). In this study, we focused only on whether or not the *m*-th gene regulates the *n*-th gene. However, the method based on the sensitivity analysis used in this study also provides us with the strength of the inferred regulation. This information would help biologists find the important regulations that are worth doing further investigation.

## Conclusion

The genetic network inference problem can be defined as a function approximation problem. On the basis of this problem definition, we proposed a new method to infer reduced NGnet models of genetic networks in this study. The experimental results on the artificial genetic network inference problems showed that the proposed method has an ability to infer genetic networks correctly. Because of the simultaneous estimation of the model parameters and the differential coefficients of the gene expression level, the models inferred by our method fitted into the observed data. Therefore, they are suitable for the computational simulation. Moreover, when trying to infer genetic networks, our method was faster than the other inference methods. As we cannot always use a high performance computer, the short computational time of our method should be a preferable feature. The proposed method was then used to analyze the SOS DNA repair system in *E. coli*, and succeeded in finding several reasonable regulations. While the number of the regulations inferred by our method was larger than that of the S-tree based method [14], its computational time was much shorter.

There does not seem to be a perfect model for the inference of genetic networks yet. Therefore, in order to extract reliable information from the observed gene expression data, the genetic networks should be inferred using a number of different models. The reduced NGnet model should be one of the promising models for this purpose.

## Methods

In this study, we propose a method to infer reduced NGnet models of genetic networks. We should note here that one model in this study corresponds to one gene. Therefore, when we try to solve a genetic network inference problem consisting of *N *genes, we must obtain *N *models, each corresponding to one of the genes. This section will describe the algorithm to obtain the reduced NGnet model corresponding to the *n*-th gene, first. Then, we will explain the technique to extract the information from the obtained models.

### Inference of a reduced NGnet model

#### Problem definition

In the genetic network inference problem, we must find a good approximation of the function *G*_*n *_(*n *= 1, ..., *N*), given in the equations (1). We define this problem as a function approximation problem in this study [7,8,10]. When trying to obtain an approximation of the function *G*_*n *_on the basis of the function approximation problem, we must give observations at *T *points

$$\left({X|}_{t_{1}},{\frac{dX_{n}}{dt}|}_{t_{1}}\right),\cdots ,\left({X|}_{t_{T}},{\frac{dX_{n}}{dt}|}_{t_{T}}\right),$$

where ${X|}_{t_{k}}=\left({X_{1}|}_{t_{k}},\cdots ,{X_{N}|}_{t_{k}}\right)$ is the expression levels of all of the genes at time *t*_*k*_, and ${\frac{dX_{n}}{dt}|}_{t_{k}}$ is the differential coefficient of the expression levels (rate of transcription) of the *n*-th gene at time *t*_*k*_. The purpose of this problem is then to estimate the parameters of the function approximator that outputs ${\frac{dX_{n}}{dt}|}_{t_{k}}(k=1,\cdots ,T)$ against a corresponding input ${X|}_{t_{k}}$.

Though DNA microarray technologies allow us to measure the gene expression levels, we have yet to find a biological technique capable of measuring the differential coefficient of the gene expression level. As an alternative, the data we obtain by measuring the time-series of the gene expression levels allow us to estimate the differential coefficients using interpolation techniques, such as the spline interpolation [32], the local linear regression [33], the neural network [8], or the Whittaker's smoother [23]. This study estimates them using the method proposed in the *Estimation of differential coefficients *section. When both the gene expression levels and their differential coefficients are given, the genetic network inference problem described here becomes solvable.

#### Reduced NGnet model

Any type of function approximator is available for approximating the function *G*_*n*_. This study however uses a reduced Normalized Gaussian network (NGnet) model [22], that was proposed by modifying an NGnet model [34,35], since we can easily estimate the model parameters using the EM algorithm. The original NGnet model, which transforms an *N*-dimensional input vector **x **to an output *y*, is defined as

(6) $$y=\sum_{i=1}^{M}\left[\frac{\alpha_{i}N(x|m_{i},\Sigma_{i})}{\sum_{j=1}^{M}\alpha_{j}N(x|m_{j},\Sigma_{j})}\right]\;\left(w_{i}\cdot x+b_{i}\right),$$

where the dot (·) denotes an operator of the inner product, and *N*(**x**|**m**_*i*_, Σ_*i*_) is an *N*-dimensional Gaussian function; its center is an *N*-dimensional vector **m**_*i *_and its covariance matrix is an (*N *× *N*)-dimensional matrix Σ_*i*_. The *N*-dimensional vector **w**_*i *_and the scalar *b*_*i *_are the linear regression parameters, *α*_*i *_($\sum_{i=1}^{M}\alpha_{i}=1$ and *α*_*i *_> 0) is the weight parameter, and *M *is the number of units. The NGnet model softly partitions the input space into *M *regions using *M *Gaussian functions. The *i*-th unit linearly approximates its output by **w**_*i*_·**x **+ *b*_*i *_within the corresponding region. The weighted sum of these outputs is the final output of the NGnet model. In the genetic network inference problem, the input vector **x **represents the expression levels of all of the genes, i.e., **X **= (*X*_1_, ..., *X*_*N*_), and the output, *y *represents the differential coefficient of the expression level of the *n*-th gene, i.e., $\frac{dX_{n}}{dt}$.

As the original NGnet, model has a large number of the model parameters, we must give a large number of observations to obtain a good function approximation. This nature is not preferable for the inference of genetic networks, since the measurement of the gene expression patterns is expensive. In order to decrease the amount of data we must observe, we limited a covariance matrix of the NGnet model Σ_*i *_to being diagonal [22]. This restricted model was referred to as the reduced NGnet model. While the number of the parameters of the original NGnet model is *O*(*N*^2^), that of the reduced model is *O*(*N*).

This study approximates the function *G*_*n *_using the reduced NGnet model, as mentioned above. Therefore, our object in this study is to find the parameters of the reduced NGnet model that outputs ${\frac{dX_{n}}{dt}|}_{t_{k}}$ (*k *= 1, ⋯, *T*) against a corresponding input ${X|}_{t_{k}}$. Our algorithm to estimate these parameters is described below.

#### Gradual reduction strategy

We cannot make the Gaussian function *N*(**x**|**m**, Σ) independent from any components of the input vector **x**, since its covariance. matrix Σ must be non-singular. As the reduced NGnet model contains several Gaussian functions, its output *y *also depends on all of the components of the input vector **x**. This fact indicates that, even when the *n*-th gene is unaffected by the *m*-th gene in the target network, the reduced NGnet model cannot capture the disconnection.

In order to overcome this drawback of the reduced NGnet model, we use the gradual reduction strategy [22]. When trying to obtain an approximation of the function *G*_*n*_, this strategy decreases the input dimension of the model by removing the genes that are assumed to unaffect the *n*-th gene. As the model obtained by this strategy has no input from the removed genes, its output is independent from these genes. In order to determine the reasonable number of the input dimension of the model, this study uses the Bayesian Information Criterion (BIC) [36], a measure for evaluating statistical models. The followings are the algorithm of the gradual reduction strategy used in this study:

1. Let a set *C *be {1, ..., *N*}, where *N *is the number of the genes in the target network. We call the genes whose indices are contained in set *C *the candidate genes. Let *N*_*C *_be the number of the elements of set *C*.

2. Extract the expression data of the candidate genes from the whole observed data. Then, obtain the reduced NGnet model by applying the DAEM algorithm, described below, to the constructed data. Note that, as the constructed data contain the expression levels of the candidate genes only, the input dimension of the model is *N*_*C*_.

3. Compute the BIC of the model obtained in step 2. The BIC of the reduced NGnet model is defined as

(7) BIC = *M*(3*N*_*C *_+ 2)log(*T*) - 2*L*,

where

(8) $$L=-\frac{T}{2}\log\left\{\frac{1}{T}\sum_{t=1}^{T}{\left[y_{t}-y(x_{t})\right]}^{2}\right\},$$

**x**_*t *_is the expression levels of the candidate genes at time *t*, *y*_*t *_is the differential coefficient of the expression level of the *n*-th gene at time *t *(i.e., ${\frac{dX_{n}}{dt}|}_{t}$) and *y*(**x**_*t*_) is the output of the obtained model against the input **x**_*t*_.

4. If the BIC calculated in step 3 is larger than of the previous iteration, output the model of the previous step and, then, stop.

5. Using the method based on the sensitivity analysis (see the *Model interpretation *section) [10], choose the genes that are assumed to unaffect the *n*-th gene. Then, remove the indices corresponding to the selected genes from set *C*. When no gene is selected, remove the index of the gene that has the weakest regulation to the *n*-th gene.

6. Return to step 2.

#### DAEM algorithm

We can use the EM algorithm to obtain the reduced NGnet model that outputs *y*_*t *_(*t *= 1, ..., *T*) against a corresponding input **x**_*t*_, since it can be interpreted as a stochastic model [9,22,37]. In our genetic network inference problem, the input **x**_*t *_and the output *y*_*t *_represent the given expression levels of the candidate genes at time *t *and the given differential coefficient of the expression level of the *n*-th gene at time *t*, respectively.

The EM algorithm however often fails to estimate reasonable model parameters because it is based on a local search. In order to enhance the probability to obtain a reasonable model, this study therefore uses the Deterministic Annealing EM (DAEM) algorithm [38], a variant of the EM algorithm. The DAEM algorithm used in this study estimates the model parameters *θ *= {*μ*_*i*_, Σ_*i*_, **w**_*i*_, *b*_*i*_, *α*_*i*_, $\sigma_{i}^{2}$|*i *= 1, ..., *M*} according to the following procedure [9,22].

1. Let *θ*^(0)^, which is generated randomly, be the initial estimate of the model parameters *θ*. Set the counter *k *and the parameter corresponding to the temperature *β *to 0 and *β*_*min*_, respectively.

2. For each pair of the input and the output (**x**_*t*_, *y*_*t*_), compute

(9) $$f_{i}^{\left(k\right)}(y_{t}|x_{t})=\frac{P{\left(x_{t},y_{t},i|\theta^{\left(k\right)}\right)}^{\beta }}{\sum_{j=1}^{M}P{\left(x_{t},y_{t},j|\theta^{\left(k\right)}\right)}^{\beta }},$$

where

(10) *P*(**x**, *y*, *i*|*θ*) = *P*(*i*|*θ*) *P*(**x**|*i*, *θ*) *P*(*y*|**x**, *i*, *θ*),

(11) *P*((*i*|*θ*) = *α*_*i*_,

(12) *P*((**x**|*i*|, *θ*) = *N *(**x**|**m**_*i*_, Σ_*i*_),

(13) $$P(y|x,i,\theta )=\frac{1}{\sqrt{2\pi \sigma_{i}^{2}}}\exp\left[-\frac{{\left(y-w_{i}\cdot x-b_{i}\right)}^{2}}{2\sigma_{i}^{2}}\right].$$

Then, form a function

(14) $$\begin{array}{l} Q_{\beta }(\theta |\theta^{\left(k\right)})\\ =\sum_{t=1}^{T}\sum_{i=1}^{M}f_{i}^{\left(k\right)}(y_{t}|x_{t})\log P(x_{t},y_{t},i|\theta )\\ +\log P_{\beta }(\theta ), \end{array}$$

where *P*_*β *_(*θ*) is a prior probability distribution. We utilize a priori knowledge about genetic networks using *P*_*β *_(*θ*), as described below.

3. Find a new estimate *θ*^(*k *+ 1) ^that maximizes the function *Q*_*β*_.

4. *k *← *k *+ 1.

5. Repeat steps 2, 3 and 4 until convergence.

6. *β *← *β *+ *β*_*add*_.

7. Stop if *β *> 1. Otherwise, return to step 2.

The parameters of the DAEM algorithm, *β*_*min *_and *β*_*add*_, were both set to 0.1 in this study.

Note that this algorithm estimates the parameter $\sigma_{i}^{2}$ along with the other model parameters. Although the reduced NGnet model does not contain the parameter, $\sigma_{i}^{2}$ we cannot derive the learning algorithm without estimating it.

#### Use of a priori knowledge

Our method may infer multiple candidate networks due to the high degree-of-freedom of the reduced NGnet model and the pollution of the observed data by the noise. In order to increase the probability to obtain a reasonable network, we therefore utilize a priori knowledge about the genetic network in this study [9]. Genetic networks are known to be sparsely connected [39]. In order to utilize this knowledge, we use the following function as *P*_*β *_(*θ*) given in the equation (14).

(15) $$P_{\beta }(\theta )=\frac{1}{Z_{\beta }}{\left[\exp\left(-\gamma T\sum_{j\in A}\sum_{i=1}^{M}w_{i,j}^{2}\right)\right]}^{\beta },$$

where *Z*_*β *_is a normalization factor, *w*_*i*,*j *_is the *j*-th component of the vector **w**_*i*_, *β *is the parameter of the DAEM algorithm, *T *is the number of the observations, *γ *is a constant parameter, and *A *is a set of indices corresponding to genes that are assumed to unaffect the *n*-th gene. The set *A *is constructed as follows.

1. Let the set *A *be {1, ..., *N*}.

2. Choose *I *genes in ascending order of the value of $\omega_{m}=\sum_{i=1}^{M}w_{i,m}^{2}$ where *I *is a parameter named the maximum indegree. The maximum indegree determines the maximum number of genes that directly affect the *n*-th gene.

3. From the set *A*, remove the indices corresponding to the genes selected in the previous step.

When the *n*-th gene is assumed to be unaffected by the *m*-th gene, this probability distribution forces the corresponding regression parameters, i.e., *w*_1,*m*_, ..., *w*_*M*,*m*_, down to zero. As mentioned in the *Gradual reduction strategy *section, even when these parameters are zero, the weak regulation of the *n*-th gene from the *m*-th gene remains in the model. However, the gradual reduction strategy should remove these unnecessary regulations from the model.

### Estimation of differential coefficients

The genetic network inference problem of this study requires estimating the differential coefficients of the gene expression levels from the observed time-series data, as mentioned in the previous section. We can use some interpolation technique to estimate them [8,15,17,23]. However, it is often difficult to estimate the differential coefficients correctly because the noise contained in the observed time-series data easily disrupts the information about their slopes. Moreover, even when these data are correctly estimated, the reduced NGnet model may not have the ability to represent them with perfect precision. As a result, when we try to simulate the gene expression using the models inferred from these data, the computed expression levels may not resemble the observed data. These models are therefore not suitable for the computational simulation. 

**Figure 7:**
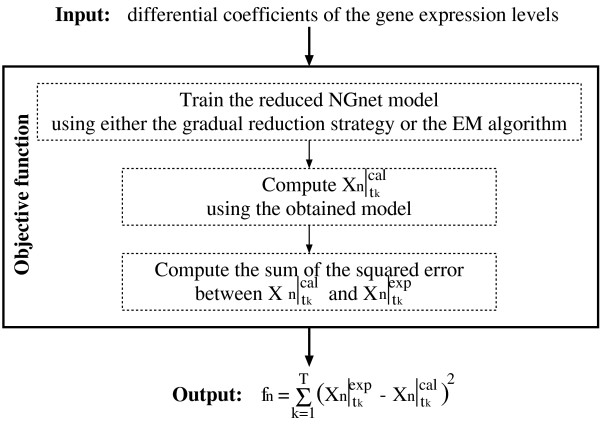
The objective function for the estimation of the differential coefficients of the *n*-th gene's expression levels.

In order to obtain the reduced NGnet models suitable for the computational simulation, we must carefully estimate the differential coefficients of the gene expression levels. We define this estimation problem as a function minimization problem in this study. The following equation is the objective function to estimate the differential coefficients of the *n*-th gene (see also Figure 7).

(16) $$f_{n}=\sum_{k=1}^{T}{\left({X_{n}|}_{t_{k}}^{\exp}-{X_{n}|}_{t_{k}}^{\text{cal}}\right)}^{2},$$

where ${X_{n}|}_{t_{k}}^{\exp}$ is the experimentally observed gene expression level of the *n*-th gene at time *t*_*k*_, and ${X_{n}|}_{t_{k}}^{\text{cal}}$ is the numerically calculated one. In order to compute ${X_{n}|}_{t_{k}}^{\text{cal}}$, we utilize the problem decomposition strategy [17,40]. In this strategy, ${X_{n}|}_{t_{k}}^{\text{cal}}$ is obtained by solving

(17) $$\frac{dX_{n}}{dt}={\hat{G}}_{n}(Y_{1},\cdots ,Y_{N}),$$

where

(18) $$Y_{m}=\{\begin{array}{ll} X_{m}, & \text{if }m=n,\\ {\hat{X}}_{m}, & \text{otherwise,} \end{array}$$

${\hat{G}}_{n}$ is the reduced NGnet mode, i.e., the right hand side of the equation (6), that approximates the function *G*_*n *_given in the equations (1), and ${\hat{X}}_{m}$ is the *m*-th gene's expression level acquired by making a direct estimation from the observed time-series data. In order to estimate ${\hat{X}}_{m}$'s, this study uses either the spline interpolation [32] for noise-free data or the local linear regression [33] for noisy data.

As mentioned above, whenever trying to compute the objective function (16), we must train the reduced NGnet model. For this purpose, we use two methods, the gradual reduction strategy described above and the EM algorithm. This study uses the former one to enhance the probability of obtaining a reasonable model and the latter to reduce the computational cost. As the EM algorithm is identical to the DAEM algorithm with *β*_*min *_= 1, its computational time is short. In our EM algorithm, in order to enhance the probability of finding a reasonable model, the parameters of the best model that has ever been found through the search are used as the initial estimate. When the function optimizer first tries to compute the objective function (16), the gradual reduction strategy is always used to infer the model. In the other cases, we use the EM algorithm with the probability 1 - 0.1 *T*^-1^, otherwise we use the gradual reduction strategy, where *T *is the number of the observations.

The dimension of the function minimization problem defined here is identical to the number of the observations *T*. Therefore, when a large amount of the data are given, we must solve very high-dimensional problems. A high-dimensional problem generally requires a high computational effort even when we use a sophisticated function optimizer. In order to reduce the computational cost, this study therefore optimizes the objective function (16) for every dimension using a one-dimensional search algorithm, the Brent's method [32].

We should note that, although the purpose of the problem described here is to obtain the reasonable differential coefficients of the gene expression levels, we can obtain the reduced NGnet model suitable for the computational simulation at the same time. Thus, this study infers a model of a genetic network by optimizing the objective function (16).

### Model interpretation

When analyzing a genetic network, we must know whether the *n*-th gene is affected by the *m*-th gene. We extract this information from the reduced NGnet model obtained using the method based on the sensitivity analysis [10].

This extraction method uses the positive and negative sensitivity coefficients averaged over time, $S_{n}^{+}(m)$ and $S_{n}^{-}(m)$, respectively. To cope with the difficulty of calculating these values precisely, the method approximates them as

(19) $$S_{n}^{+}(m)≃\frac{1}{T}\sum_{k=1}^{T}h\left({\frac{\partial {\hat{G}}_{n}}{\partial X_{m}}|}_{t_{k}}\right),$$

and

(20) $$S_{n}^{-}(m)≃\frac{1}{T}\sum_{k=1}^{T}h\left(-{\frac{\partial {\hat{G}}_{n}}{\partial X_{m}}|}_{t_{k}}\right),$$

where

(21) $$h(x)=\{\begin{array}{ll} x, & \text{if }x>0,\\ 0, & \text{otherwise,} \end{array}$$

*T *is the number of sampling points of the measured time-series data, and ${\frac{\partial {\hat{G}}_{n}}{\partial X_{m}}|}_{t}$ is the estimated sensitivity coefficient at time *t*, that is calculated from the reduced NGnet model obtained. As the model used in this study is differentiable, we can calculate ${\frac{\partial {\hat{G}}_{n}}{\partial X_{m}}|}_{t}$ analytically.

As the sensitivity coefficients represent the impact of the *m*-th gene upon the *n*-th gene, the large values of $S_{n}^{+}(m)$ and $S_{n}^{-}(m)$ indicate the positive and negative regulations, respectively, of the *n*-th gene from the *m*-th gene. Therefore, the method used in this study concludes that the *n*-th gene is positively regulated by the *m*-th gene when $S_{n}^{+}(m)+S_{n}^{-}(m)$ exceeds a threshold *Thresh*(*n*) and

(22) $$\frac{S_{n}^{+}(m)}{S_{n}^{+}(m)+S_{n}^{-}(m)}>a,$$

where *a *is a constant parameter. Similarly, when $S_{n}^{+}(m)+S_{n}^{-}(m)$ > *Thresh*(*n*) and

(23) $$\frac{S_{n}^{-}(m)}{S_{n}^{+}(m)+S_{n}^{-}(m)}>a,$$

the method infers the negative regulation of the *n*-th gene from the *m*-th gene. Conversely, when $S_{n}^{+}(m)+S_{n}^{-}(m)$ ≤ *Thresh*(*n*), no regulation of the *n*-th gene from the *m*-th gene is inferred. According to the reference [10], we set

(24) $$Thresh(n)=b\underset{m}{\max}\left[S_{n}^{+}(m)+S_{n}^{-}(m)\right],$$

*a *= 0.3 and *b *= 0.05. Note that, when the *m*-th gene promotes both of the synthesis and the degradation of the *n*-th gene for example, the values of $S_{n}^{+}(m)$ and $S_{n}^{-}(m)$ might be large. In these cases, the extraction method must infer both of the positive and the negative regulations of the *n*-th gene from the *m*-th gene. As *a *is set to less than 0.5, the method has an ability to infer these regulations simultaneously.

## Authors' contributions

SK designed the inference method and performed the experiments. KS and SY proposed a basic idea of the inference method. HM and KM implemented some parts of the proposed algorithm. MH supervised the biological aspect of this work. All authors read and approved the manuscript.

## Supplementary Material

Additional file 1 This file provides supplementary information. Click here for file

## References

[B1] Akutsu T, Miyano S, Kuhara S (2000). Inferring Qualitative Relations in Genetic Networks and Metabolic Pathways. Bioinformatics.

[B2] D'haeseleer P, Liang S, Somogyi R (2000). Genetic Network Inference: From Co-expression Clustering to Reverse Engineering. Bioinformatics.

[B3] Imoto S, Goto T, Miyano S (2002). Estimation of Genetic Networks and Functional Structures between Genes by using Bayesian Network and Nonparametric Regression. Proc Pacific Symposium on Biocomputing.

[B4] Maki Y, Tominaga D, Okamoto M, Watanabe S, Eguchi Y (2001). Development of a System for the Inference of Large Scale Genetic Networks. Proc Pacific Symposium on Biocomputmg.

[B5] Sakamoto E, Iba H (2001). Inferring a System of Differential Equations for a Gene Regulatory Network by using Genetic Programming. Proceedings of the 2001 Congress on Evolutionary Computation: 2001; Seoul.

[B6] Vance W, Arkin A, Ross J (2002). Determination of Causal Connectivities of Species in Reaction Networks. Proc Natl Acad Sci USA.

[B7] Yeung MKS, Tegnér J, Collins JJ (2002). Reverse Engineering Gene Networks using Singular Value Decomposition and Robust Regression. Proc Natl Acad Sci USA.

[B8] Voit EO, Almeida J (2004). Decoupling Dynamical Systems for Pathway Identification from Metabolic Profiles. Bioinformatics.

[B9] Kimura S, Sonoda K, Yamane S, Matsumura K, Hatakeyama M (2006). Function Approximation Approach to the Inference of Normalized Gaussian Network Models of Genetic Networks. Proceedings of the 2006 International Joint Conference on Neural Networks: 2006; Vancouver.

[B10] Kimura S, Sonoda K, Yamane S, Matsumura K, Hatakeyama M (2007). Function Approximation Approach to the Inference of Neural Network Models of Genetic Networks. IPSJ Transactions on Bioinformatics.

[B11] Bernardo D, Thompson MJ, Gardner TS, Chobot SE, Eastwood EL, Wojtovich AP, Elliott SJ, Schaus SE, Collins JJ (2005). Chemogenomic Profiling on a Genome-wide Scale using Reverse-engineered Gene networks. Nature Biotechnology.

[B12] Gardner TS, Bernardo D, Lorenz D, Collins JJ (2003). Inferring Genetic Networks and Identifying Compound Mode of Action via Expression Profiling. Science.

[B13] Voit EO (2000). Computational Analysis of Biochemical Systems.

[B14] Cho DY, Cho KH, Zhang BT (2006). Identification of Biochemical Networks by S-tree Based Genetic Programming. Bioinformatics.

[B15] Chou IC, Martens H, Voit EO (2006). Parameter Estimation in Biochemical Systems Models with Alternating Regression. Theoretical Biology and Medical Modelling.

[B16] Kikuchi S, Tominaga D, Arita M, Takahashi K, Tomita M (2003). Dynamic Modeling of Genetic Networks using Genetic Algorithm and S-system. Bioinformatics.

[B17] Kimura S, Hatakeyama M, Konagaya A (2004). Inference of S-system Models of Genetic Networks from Noisy Time-series Data. Chem-Bio Informatics J.

[B18] Kimura S, Ide K, Kashihara A, Kano M, Hatakeyama M, Masui R, Nakagawa N, Yokoyama S, Kuramitsu S, Konagaya A (2005). Inference of S-system Models of Genetic Networks using a Cooperative Coevolutionary Algorithm. Bioinformatics.

[B19] Kutalik Z, Tucker W, Moulton V (2007). S-system Parameter Estimation for Noisy Metabolic Profiles using Newton-flow Analysis. IET Systems Biology.

[B20] Tominaga D, Horton P (2006). Inference of Scale-free Networks from Gene Expression Time Series. J of Bioinformatics and Computational Biology.

[B21] Tsai KY, Wang FS (2005). Evolutionary Optimization with Data Collocation for Reverse Engineering of Biological Networks. Bioinformatics.

[B22] Kimura S, Sonoda K, Yamane S, Yoshida K, Matsumura K, Hatakeyama M (2007). Inference of Genetic Networks using a Reduced NGnet Model. Proceedings of the 2007 International Joint Conference on Neural Networks: 2007: Orlando.

[B23] Vilela M, Borges CCH, Vinga S, Vanconcelos ATR, Santos H, Voit EO, Almeida JS (2007). Automated Smoother for the Numerical Decoupling of Dynamics Models. BMC Bioinformatics.

[B24] Savageau MA (1969). Biochemical Systems Analysis I. Some Mathematical Properties of the Rate Law for the Component Enzymatic Reactions. J Theoret Biol.

[B25] Shiraishi F, Savageau MA (1992). The Tricarboxylic Acid Cycle in Dictyostelium Discoideum. J of Biological Chemistry.

[B26] Voit EO, Radivoyevitch T (2000). Biochemical Systems Analysis of Genome-wide Expression Data. Bioinformatics.

[B27] Hlavacek WS, Savageau MA (1996). Rules for Coupled Expression of Regulator and Effector Genes in Inducible Circuits. J Mol Biol.

[B28] Veflingstad SR, Almeida J, Voit EO (2004). Priming Nonlinear Searches for Pathway Identification. Theoretical Biology and Medical Modelling.

[B29] Sutton MD, Smith BT, Godoy VG, Walker GC (2000). The SOS Response: Recent Insights into umuDC-Dependent Mutagenesis and DNA Damage Tolerance. Annual Review of Genetics.

[B30] Ronen M, Rosenberg R, Shraiman BI, Alon U (2002). Assigning Numbers to the Arrows: Parameterizing a Gene Regulation Network by using Accurate Expression Kinetics. Proc Natl Acad Sci USA.

[B31] Data of SOS system reporter plasmids. http://www.weizmann.ac.il/mcb/UriAlon/Papers/SOSData/.

[B32] Press WH, Teukolsky SA, Vetterling WT, Flannery BP (1995). Numerical Recipes in C.

[B33] Cleveland WS (1979). Robust Locally Weight Regression and Smoothing Scatterplots. J of American Statistical Association.

[B34] Moody J, Darken CJ (1989). Fast Learning in Networks of Locally-tuned Processing Units. Neural Computation.

[B35] Sato M, Ishii S (2000). On-line EM Algorithm for the Normalized Gaussian Network. Neural Computation.

[B36] Schwarz G (1978). Estimating the Dimension of a Model. Annals of Statistics.

[B37] Xu L, Jordan MI, Hinton GE (1995). An Alternative Model for Mixtures of Experts. Advances in Neural Information Processing Systems.

[B38] Ueda N, Nakano R (1998). Deterministic Annealing EM Algorithm. Neural Networks.

[B39] Thieffry D, Huerta AM, Pérez-Rueda E, Collado-Vides J (1998). From Specific Gene Regulation to Genomic Networks: a Global Analysis of Transcriptional Regulation in Escherichia Coli. BioEssays.

[B40] Maki Y, Ueda T, Okamoto M, Uematsu N, Inamura Y, Eguchi Y (2002). Inference of Genetic Network using the Expression Profile Time Course Data of Mouse P19 Cells. Genome Informatics.

