# Supplementary material for: Function approximation approach to the inference of reduced NGnet models of genetic networks
Source: BMC Bioinformatics. 2008 Jan 14;9:23. doi: 10.1186/1471-2105-9-23 (PMC2258286; doi:10.1186/1471-2105-9-23)
Supplement: Additional file 1 — This file provides supplementary information. [file 1471-2105-9-23-S1.pdf]

Supplementary Information: Function Approximation Approach to the Inference of Reduced NGnet Models of Genetic Networks

Shuhei Kimura *et al.*
kimura@ike.tottori-u.ac.jp

1 Additional Experiments

1.1 Inference of a large-scale S-system model

In order to determine several hyper-parameters of the proposed method, we performed the experiment. As a target network that we attempt to infer, we used an S-system model consisting of 30 genes ($N = 30$). The network structure and the S-system parameters of the target network are shown in Figure 1 and Table 1, respectively [4]. As the observed gene expression patterns, fifteen sets of noise-free time-series data, each covering all 30 genes, were given. The sets began from randomly generated initial values in $[0.0, 2.0]$ and were obtained by solving the differential equations (2) on the target model. Eleven sampling points for the time-series data were assigned to each gene in each set. We set the maximum indegree I to 5, and all of the other experimental conditions were the same as those used in the original paper.

Figure 2 shows a typical genetic network inferred from the obtained models. As the figure illustrates, most of the regulations were correctly inferred by the proposed method. Our method inferred an average of 2.4 ± 2.6 false-positive regulations, and failed to infer an average of 1.4 ± 1.2 regulations (false-negative regulations). The sensitivity and the specificity of the proposed method were therefore 0.979 ± 0.018 and 0.999 ± 0.001, respectively. The value of the objective function (16) was $1.49 \times 10^{-2} \pm 1.37 \times 10^{-1}$ on average. The proposed method required about $3.85 \times 30 \simeq 115$ min to solve this genetic network inference problem on a single-CPU personal computer (Pentium IV 2.8GHz).

The proposed method contains several hyper-parameters we must determine. The performances of our method should depend on these parameters. Therefore, we must carefully choose their values when we apply it to genetic network inference problems. Figure 3(a), (b) and (c) show the sensitivity and the specificity of the proposed method on the experiments where the different values of the weight parameter γ, the maximum indegree I and the number of the units M, respectively, were used. The figures indicate that the performances of our method have a peak nearly at the parameter values used in this study, i.e., $\gamma = 20$, $I = 5$ and $M = 3$.

1.2 Inference of a random genetic network of 30 genes

In order to describe genetic networks, our method uses a reduced NGnet model that was proposed by modifying an NGnet model. Therefore, we compared the proposed method with an inference method of using the NGnet model. The target networks, consisting of 30 genes ($N = 30$), were the same as those used in the *Inference of a random genetic network* section of the original paper.

Figure 4(a) and (b) illustrate the sensitivities and the specificities, respectively, of the proposed method and the inference method of using the original NGnet model on the experiments where different numbers of time-series sets were given. As the figures show, the performances of the two methods increased with an increase in the amount of the observed data. This result suggests that a sufficient amount of the data enables us to infer a reasonable genetic network even when they are polluted by noise. Although the reference [22] has demonstrated the effectiveness of the use of the reduced NGnet model, the experimental results in this study indicate that the proposed method did not always outperform the inference method based on the original NGnet model. In order to determine the reasonable number of the regulations contained in the inferred network, this study uses the Bayesian Information Criterion (BIC) [32], a measure for trading off the number of the model parameters against how well the model fits the data. As the original NGnet model contains a larger number of the parameters, the BIC should cause the inferred network containing a smaller number of the regulations. The smaller number of the inferred regulations is one of the reasons that the specificity of the method based on the original NGnet model was better than that of the proposed method.

Table 1: The parameters of the large-scale target model.

α_n	1.0
β_n	1.0
$g_{n,m}$	$g_{1,14} = -0.1,\ g_{5,1} = 1.0,\ g_{6,1} = 1.0,\ g_{7,2} = 0.5,$ $g_{7,3} = 0.4,\ g_{8,4} = 0.2,\ g_{8,17} = -0.2,\ g_{9,5} = 1.0,$ $g_{9,6} = -0.1,\ g_{10,7} = 0.3,\ g_{11,4} = 0.4,\ g_{11,7} = -0.2,$ $g_{11,22} = 0.4,\ g_{12,23} = 0.1,\ g_{13,8} = 0.6,\ g_{14,9} = 1.0,$ $g_{15,10} = 0.2,\ g_{16,11} = 0.5,\ g_{16,12} = -0.2,\ g_{17,13} = 0.5,$ $g_{19,14} = 0.1,\ g_{20,15} = 0.7,\ g_{20,26} = 0.3,\ g_{21,16} = 0.6,$ $g_{22,16} = 0.5,\ g_{23,17} = 0.2,\ g_{24,15} = -0.2,\ g_{24,18} = -0.1,$ $g_{24,19} = 0.3,\ g_{25,20} = 0.4,\ g_{26,21} = -0.2,\ g_{26,28} = 0.1,$ $g_{27,24} = 0.6,\ g_{27,25} = 0.3,\ g_{27,30} = -0.2,\ g_{28,25} = 0.5,$ $g_{29,26} = 0.4,\ g_{30,27} = 0.6,$ other $g_{n,m} = 0.0$
$h_{n,m}$	1.0 if $n = m$, 0.0 otherwise.

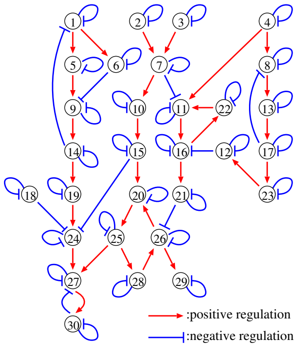

Figure 1: The network structures of the large-scale target model.

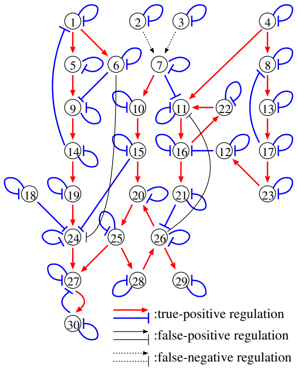

Figure 2: A sample of the network structure inferred by the proposed method.

We can however easily improve the specificity of the proposed method by means of removing the weak regulations from the inferred network.

2 Supplementary Data

2.1 Actual gene expression data

Figure 5 shows the observed data of the SOS DNA repair system used in the *Inference of an actual genetic network* section. Two sets of the time-series data of 6 genes were used.

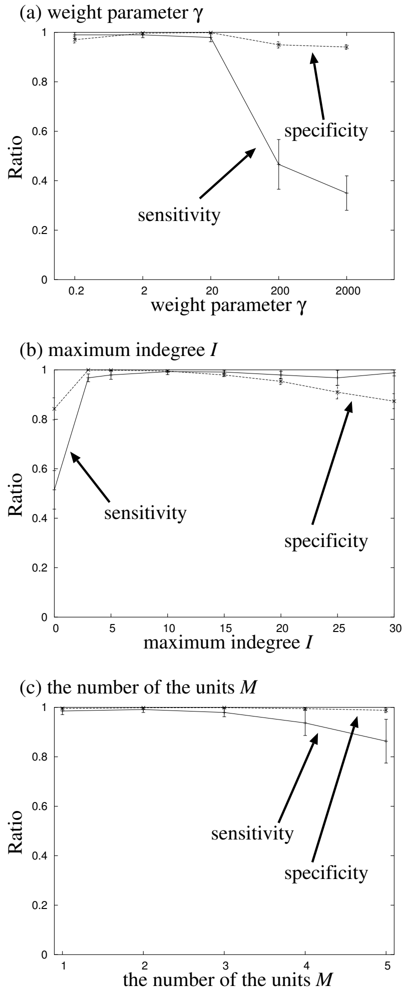

Figure 3: The performances of the proposed method on the experiments where the different values of (a) the weight parameter γ, (b) the maximum indegree I and (c) the number of the units M, respectively, were used. Solid line: the sensitivity. Dotted line: the specificity.

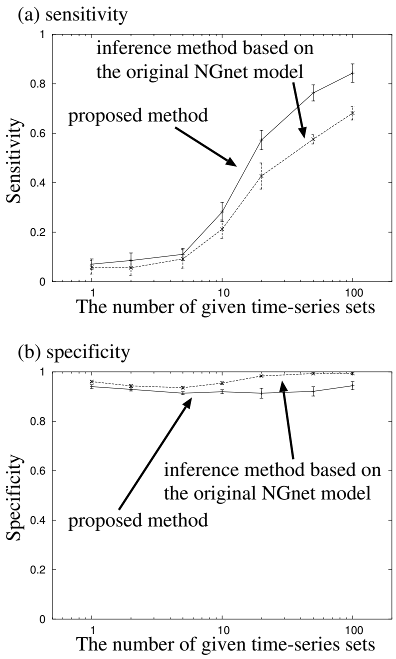

Figure 4: The performances of the proposed method based on the reduced NGnet model (solid line) and the inference method based on the original NGnet model (dotted line). The figures show (a) the sensitivity and (b) the specificity of the two methods.

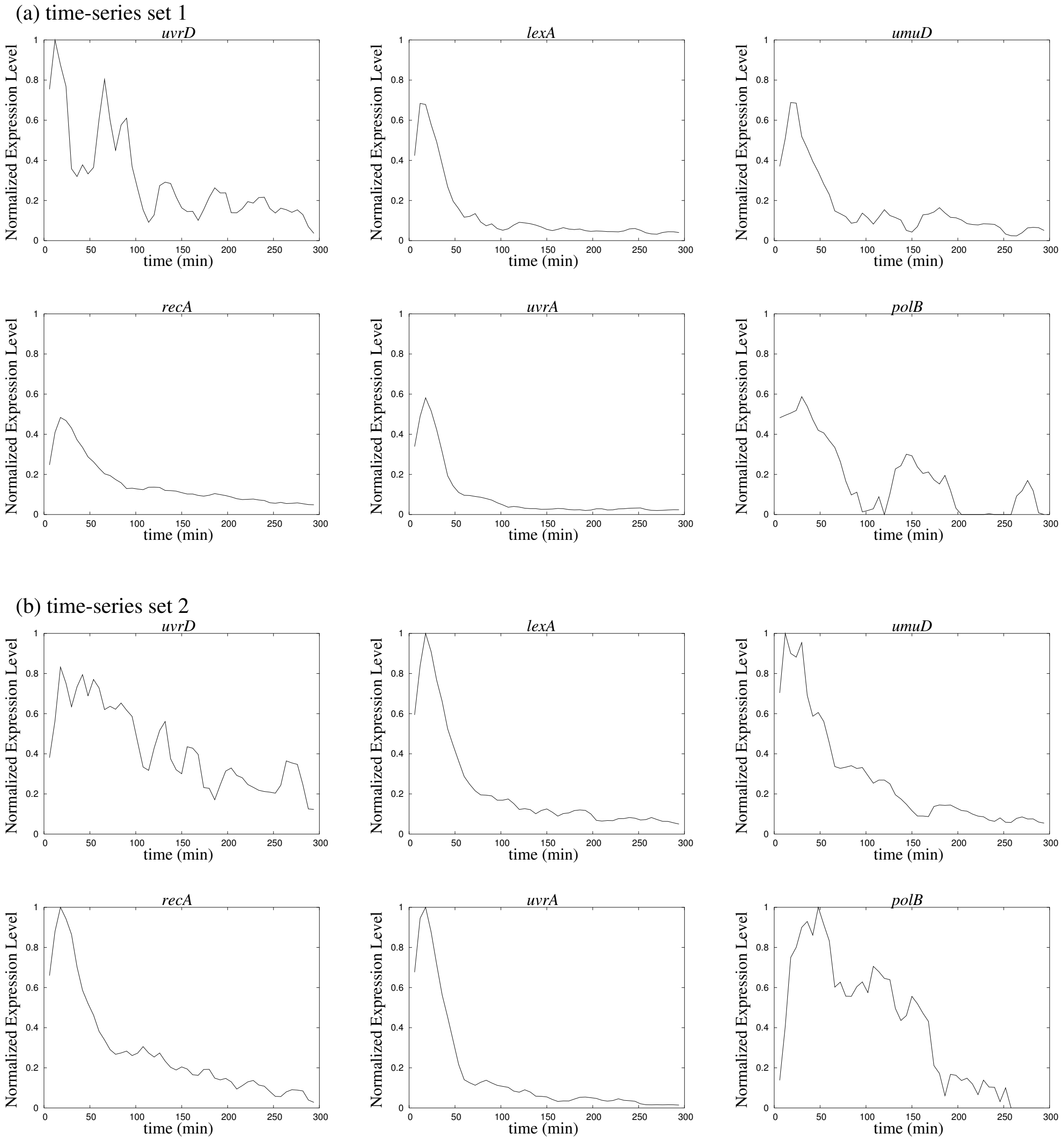

Figure 5: Two sets of the observed time-series data of the SOS DNA repair system used in the *Inference of an actual genetic network* section.
